# Organizational structure, worker participation, and health: a cross-sectional survey study among Japanese hospital employees

**DOI:** 10.1186/s12913-026-14786-7

**Published:** 2026-05-25

**Authors:** Nicholas M. Raposo, Åsa Kneck, Anders Kassman, Johan Vamstad, Anna Klarare

**Affiliations:** 1 Connell School of Nursing, Boston College, 140 Commonwealth Avenue, Chestnut Hill, MA 02467 USA; 2 Department of Health Care Sciences, Marie Cederschiöld University, Stockholm, Sweden; 3 Department of Civil Society and Religion, Marie Cederschiöld University, Stockholm, Sweden; 4 Department of Women’s and Children’s Health, CIRCLE, Uppsala University, Uppsala, Sweden

**Keywords:** Employee health, Worker participation, Workplace environment, Organizational culture, Personnel administration, Hospital, Japan

## Abstract

**Background:**

Worker participation, reflecting perceptions of control and influence in one’s work environment, is known to buffer job stress and promote employee wellbeing, but opportunities for participation are influenced by organizational dynamics. In Japan, hospitals operate under various organizational structures characterized by distinct governance, culture, and values. How these structural differences shape worker participation and, ultimately, health remains unclear. The purpose of this study was to explore how organizational structure influences worker participation and the subsequent impact on perceived health among Japanese hospital employees.

**Methods:**

Cross-sectional survey data were collected from 6,859 employees across 10 hospitals representing three organizational structures: Japanese Health and Welfare Co-operative Federation, Japan Agricultural Cooperatives Welfare Union, and public hospitals. Worker participation was measured using two indices: (1) discretion over work, and (2) opportunities to express opinions. Self-rated health was assessed using a single item. Kruskal-Wallis tests were used to compare worker participation scores across organizational structures and self-health ratings. Then, ordinal logistic regression was used to estimate the odds of better self-rated health per unit increase in participation index scores. Finally, multilevel modeling was used to analyze the association between health and participation, adjusting for demographic and work-related covariates and accounting for clustering by organizational structure.

**Results:**

Worker participation scores varied significantly by organizational structure; they were highest in the more cooperative structures and lowest in the more hierarchical public structure. Each one-point increase in Index 1 and Index 2 scores was associated with 38% (OR = 1.38, 95% CI = 1.28–1.49, *p* < 0.001) and 49% (OR = 1.49, 95% CI = 1.39–1.60, *p* < 0.001) higher odds of better self-rated health, respectively. Notably, the positive association between worker participation and health was largely consistent across organizational structures, with organizational structure contributing minimally (ICC = 0.004, 95% CI = 0.001–0.027) to self-rated health beyond worker participation.

**Conclusions:**

Organizational structure shapes worker participation, which in turn positively predicts self‑rated health among Japanese hospital employees. Policies and practices that foster participatory work environments, including enhancing employee discretion and voice, offer a practical and scalable approach to improving healthcare workforce wellbeing across varied settings.

**Supplementary Information:**

The online version contains supplementary material available at 10.1186/s12913-026-14786-7.

## Introduction

Healthcare workers’ health and wellbeing are essential for quality patient care, workforce sustainability, and organizational resilience [1, 2]. Among the approaches with the greatest potential to enhance healthcare worker wellbeing is expanding opportunities for worker participation [3]. Worker participation refers to the degree of self-determination over one’s job, including autonomy over work tasks, the use and development of skills, and engagement in decision-making processes [4]. Greater participation buffers against job stress and promotes employee engagement, satisfaction, and wellbeing [5–8].

As a feature of the work environment, worker participation is shaped by organizational dynamics like governance, culture, and values [4, 6, 9]. In Japan, where hospitals operate under several distinct organizational structures [10], it is unclear how these structural differences influence worker participation and, in turn, employee health. This study addresses this gap by exploring associations between organizational structure, worker participation, and self-rated health among hospital employees in Japan, with the aim to inform strategies to improve workforce wellbeing across varied healthcare work environments.

## Background

### The job demand-control model

Employees’ perceptions of control and influence within the work environment are central to their wellbeing. A foundational framework for understanding this relationship is Karasek’s Job Demand-Control (JDC) model [4], later developed into the Job Demand-Control-Support (JDCS) model [11]. The model posits that job strain results from high physical and psychological demands with low decision latitude (participation) and limited social support. Karasek theorized that job strain influences individual behaviors on and off the job, ultimately affecting employee wellbeing [4]. In support of this theory, he linked job strain to outcomes such as exhaustion, depression, pharmaceutical sedative use, and absenteeism.

Importantly, Karasek hypothesized that decision latitude is closely tied to an organization’s authority structure [4]. He suggested that organizational features like hierarchy, flexibility, and equity may either constrain or enhance opportunities for worker participation. However, empirical studies testing this hypothesis, particularly in healthcare, are limited.

### Organizational structure, worker participation, and health

Employee participation in the design, organization, and management of work benefits both individuals and organizations. Generally, greater participation reduces harmful work stressors, enhances workplace wellbeing and job satisfaction, and boosts productivity and service quality [12–16]. In healthcare specifically, lower participation is linked to burnout and intentions to leave [17–19], while higher participation predicts greater satisfaction and engagement [20]. Worker participation (operationalized as job control) has also been associated with health-related outcomes, including fertility [21], hypertension [22], and vegetable and alcohol consumption [23]. Evidence from Japan similarly highlights the health and occupational effects of worker participation. For example, higher participation corresponds to better job performance among civil servants [24], lower turnover in the financial sector [25], and reduced job stress among multisector employees [26]. In Japanese healthcare settings, worker participation predicts nurses’ intentions to leave their organizations and the profession [19].

As Karasek proposed, organizational dynamics seem to shape opportunities for worker participation [4]. While people-centered organizational cultures facilitate employee involvement [6], the hierarchical structures typical in healthcare settings tend to constrain workers’ voice [9]. In Japan, research has shown variation in worker participation among nurses working in hospitals, home healthcare facilities, and nursing homes [19]. However, the exact relationship between worker participation and employee health remains unclear in the Japanese healthcare context.

### Healthcare organizational structures in Japan

Japan’s healthcare system comprises an array of organizations, including public and non-profit cooperative entities, which differ in governance, workplace culture, service orientation, and social values [10]. Three major organizations reflect these distinctions: the Japanese Health and Welfare Co-operative Federation (HeW Co-Op), Koseiren, and the public hospital sector.

HeW Co-Op, the medical branch of the Japanese Consumers’ Co-operative Union, oversees a nationwide network of healthcare facilities, from large hospitals to small clinics and long-term care centers [10]. The organization espouses participatory values such as patient involvement, social entrepreneurship, and community co-creation, alongside broader social goals like peace, gender equality, and ecological sustainability [27, 28]. Employees often serve as cooperative members, participating in volunteerism and outreach.

Koseiren, the medical branch of the Japan Agricultural Cooperatives Welfare Union, generally manages larger hospitals with hierarchical, top-down governance [10]. While sharing some commitment to community-building, Koseiren facilities tend to prioritize technical and material advancement, such as acquiring cutting-edge medical equipment. Nevertheless, Koseiren employees also participate in community health promotion and voluntary outreach beyond routine clinical care [29–31].

Japan’s public healthcare sector is characterized by large, research-intensive hospitals with formal hierarchical structures [10]. Providers in these settings are highly professionalized and tend to view their jobs as careers rather than personal callings. Compared to the cooperative organizations, the public hospitals place less emphasis on social values and community engagement [31].

Together, these organizational structures foster substantial variation in workplace dynamics, culture, and values. Yet it remains unclear how these structural differences shape worker participation and, in turn, employee health across Japanese hospital settings.

### Purpose

The purpose of this study was to explore how three distinct organizational structures influence worker participation among Japanese hospital employees and the subsequent impact on their perceived health.

**Research Questions**

1. How does worker participation among Japanese hospital employees vary across the three organizational structures?
2. What is the relationship between worker participation and self-rated health among hospital employees in these settings?

## Methods

### Design

This study used a quantitative, cross-sectional survey design, reported according to Strengthening the Reporting of Observational Studies in Epidemiology (STROBE) guidelines [32]. The survey was conducted as part of a larger project examining healthcare delivery in Japanese cooperative and public hospitals. The project was led by a team of Japanese and Swedish researchers supported by Osaka University’s International Joint Research Program [10]. The primary aim was to compare healthcare delivery across cooperative and public hospital settings.

### Setting and participants

Between 2016 and 2017, survey data were collected from ten Japanese hospitals, each representing one of three organizational structures.^10^ Hospitals were purposively selected based on size (≥ 300 employees) and geographic diversity, including both small towns and large urban areas. Only hospitals employing ≥ 300 staff were included to ensure organizational comparability, as smaller clinics did not consistently offer all services examined. Participation was voluntary, anonymous, and open to all employees within these hospitals.

### Instrument and measures

The survey was designed to evaluate the work environment, working conditions, workplace social contributions, and employee health. Survey items were drawn from established sources, including the Swedish Work Environment Study [33]. A team of Japanese and Swedish researchers reviewed and adapted the Swedish Work Environment Study framework for cultural relevance, translated the instrument into Japanese, and developed a machine-readable paper questionnaire. Following translation, cultural adaptation occurred in three stages: alignment of items with Japanese hospital contexts (e.g., occupational categories), addition of context-specific examples informed by fieldwork, site visits, and institutional documentation, and independent cross-cultural review by Japanese researchers. This process led to revision or exclusion of conceptually incongruent items, including certain attitudinal questions and sensitive background variables such as income.

### Organizational structure

Participants were categorized based on the organizational structure (HeW Co-Op, Koseiren, Public) of their employing hospital.

### Worker participation

Worker participation was measured using two indices (Supplementary File 1). Index 1, discretion over work, included four items and demonstrated good internal consistency (Cronbach’s α = 0.855). Index 2, opportunities to express opinions, comprised three items with excellent internal consistency (Cronbach’s α = 0.912). All items were rated on a five-point Likert scale from disagree to agree. Responses were quantified and averaged to generate composite scores ranging from 0 to 4 per index, with higher scores indicating greater worker participation.

### Self-rated health

Self-rated health was assessed by a single item: “How would you rate your current health?” with response options: not healthy, somewhat not healthy, and healthy.

### Covariates

Participant demographic and work-related data were also collected. Variables included age (in years), sex (female or male), profession (doctor, nurse, care worker, administrator, other medical specialist, other support staff), employment status (full-time permanent, part-time temporary, fixed-term contract, commissioned staff, dispatched staff), and tenure at the current hospital (< 1 year, 1–5 years, 5–10 years, > 10 years).

### Data collection

Data collection was coordinated with hospital leaders, who informed employees about the study. A designated contact person at each hospital distributed the questionnaires, which were returned in sealed envelopes and sent to an independent data processing company for data entry and anonymization. The anonymized dataset was then provided to the research team for analysis.

### Data analysis

Descriptive statistics were calculated for all variables. Sample characteristics were summarized overall and by organizational structure. Summaries included frequencies and percentages for categorical variables and means and standard deviations for continuous variables. Differences in sample characteristics by organizational structure, worker participation, and self-rated health were examined to contextualize the sample and identify group patterns. Missingness and assumptions underlying subsequent analyses were also evaluated.

To address the first research question, Kruskal-Wallis tests were used to compare worker participation index scores across organizational structures. Post-hoc pairwise comparisons using Dunn’s method with Bonferroni correction for multiple tests were conducted to identify specific group differences. For the second research question, a two-step approach was employed: first, Kruskal-Wallis tests were run to compare worker participation index scores across levels of self-rated health, followed by post-hoc tests using Dunn’s method with Bonferroni correction. Next, unadjusted ordinal logistic regression was used to estimate odds ratios for better self-rated health per one-unit increase in worker participation scores.

Finally, multilevel ordinal logistic regression was used to model the association between worker participation and self-rated health, adjusting for covariates and accounting for clustering by organizational structure. The intraclass correlation coefficient (ICC) was calculated to quantify variance in self-rated health attributable to organizational structure. All analyses were conducted with Stata 18.0, with statistical significance set at *p* < 0.05 and adjusted for multiple comparisons as described.

### Power analysis

An a priori power analysis was conducted to determine the minimum sample size for the planned multiple regression analysis. Assuming a small effect size (*f*^2^ = 0.02), significance level of 0.05, power of 0.8, and eight predictors, at least 755 participants were required.

## Results

Of the 9,510 surveys distributed, 6,859 (72%) were returned. Response rates varied by organizational structure: 63% for HeW Co-Op, 86% for Koseiren, and 73% for Public. Overall, 92% of records were complete and key study variables were missing completely at random, supporting the use of complete case analysis.

### Descriptive statistics

#### Sample characteristics

Overall sample characteristics are presented in Table 1. The majority of respondents were female (79%) with a mean age of 41 (± 12) years. Nurses constituted the largest professional group (41%) and doctors the smallest (5%). Most participants were full-time permanent employees (68%) and had worked at their current hospitals for more than five years (56%).

**Table 1:** Characteristics of the sample overall and by organizational structure

Characteristic	Overall			HeW Co-Op			Koseiren			Public			
	*n*	%		*n*	%		*n*	%		*n*	%		*p*
*N*	6859	100		2852	41.6		2562	37.4		1445	21.1		
Sex													< 0.001
Female	5293	78.8		2225	80.0		1896	75.5		1172	82.4		
Male	1422	21.2		555	20.0		616	24.5		251	17.6		
Profession													< 0.001
Doctor	349	5.1		67	2.4		180	7.1		102	7.1		
Nurse	2773	40.6		748	26.4		1217	47.7		808	56.2		
Care Worker	1070	15.7		955	33.7		107	4.2		8	0.6		
Administrator	1101	16.1		456	16.1		365	14.3		280	19.5		
Other medical specialist	1144	16.8		481	17		457	17.9		206	14.3		
Other support staff	388	5.7		130	4.6		223	8.7		35	2.4		
Employment status													< 0.001
Full-time permanent	4593	67.5		1619	57.1		1979	77.7		995	69.9		
Part-time temporary	1526	22.4		1056	37.2		246	9.7		224	15.7		
Fixed-term contract	397	5.8		117	4.1		168	6.6		112	7.9		
Commissioned staff	183	2.7		39	1.4		83	3.3		61	4.3		
Dispatched staff	108	1.6		4	0.1		72	2.8		32	2.2		
Current hospital tenure													< 0.001
<1 year	770	11.3		341	12.1		231	9.1		198	13.8		
1–5 years	2197	32.3		965	34.1		749	29.5		483	33.7		
5–10 years	1508	22.2		680	24.1		537	21.1		291	20.3		
>10 years	2327	34.2		840	29.7		1026	40.3		461	32.2		
	*M*	*SD*		*M*	*SD*		*M*	*SD*		*M*	*SD*		
Age (years)	41	12.1		43.7	12.1		39	11.9		39.3	11.4		< 0.001

Note. Abbreviations: M, mean; SD, standard deviation

### Organizational structure

The largest proportion of respondents were from HeW Co-Op (42%) and the fewest from Public (21%). Sample characteristics varied significantly by organizational structure (Table 1). Compared to other organizational structures, HeW Co-Op respondents tended to be older, more often care workers, and more frequently held part-time temporary positions. Koseiren and Public respondents were younger and included higher proportions of doctors, nurses, and full-time permanent staff. Tenure distributions varied. Further details are presented in Table 1.

### Worker participation

Mean worker participation scores were 2.15 (± 0.93; median = 2.25) for Index 1 (discretion over work) and 2.26 (± 0.97; median = 2.33) for Index 2 (opportunities to express opinions). Worker participation scores varied across most sample characteristics (Supplementary Table 1). Age correlated with Index 1, but not Index 2, scores. Notably, doctors and male respondents scored highest on both indices, while nurses and other support staff scored lowest. Part-time temporary staff had higher Index 1 scores, whereas full-time permanent staff had higher Index 2 scores. Participation scores also varied with hospital tenure: Index 1 was lowest among those with less than one year of experience, while Index 2 was highest among those with over ten years’ experience. Supplementary Table 1 provides additional details.

### Self-rated health

Most respondents (64%) perceived they were healthy, while a minority (3%) reported they were not healthy. Self-rated health was associated with all sample characteristics except sex (Supplementary Table 2). Intuitively, self-rated health declined with age and hospital tenure. Doctors and administrators were more likely, and nurses and care workers less likely, to report being healthy. Fewer full-time permanent staff, and more part-time temporary staff, rated healthy than expected. See Supplementary Table 2 for further details.

### Key findings

#### Differences in worker participation between organizational structures

**Fig. 1:**
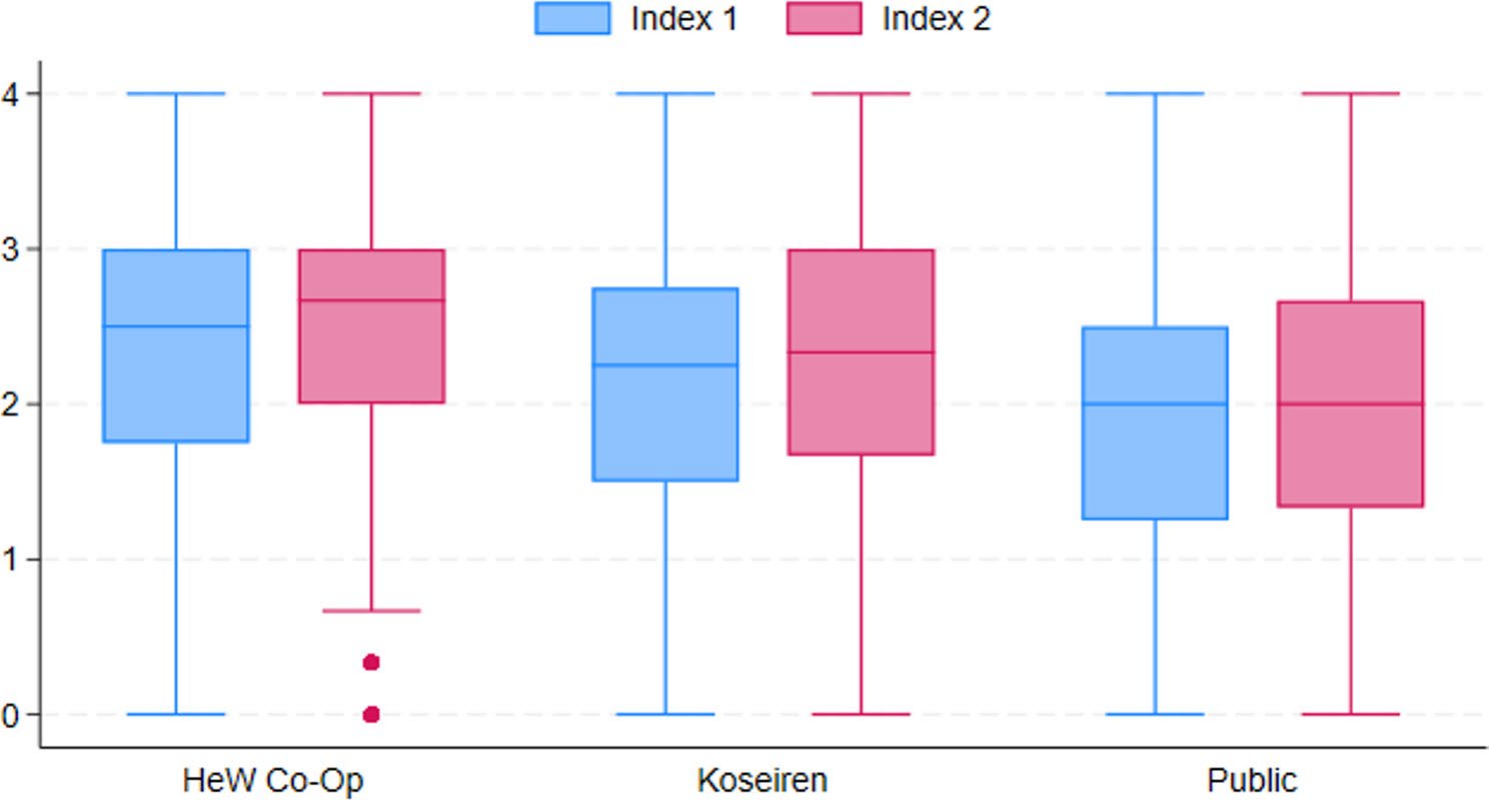
Worker participation scores by organizational structure

Distributions of worker participation scores by organizational structure are depicted in Fig. 1. The Kruskal-Wallis test indicated a significant difference in Index 1 (discretion over work) scores across organizational structures: χ^2^(2) = 215.24, *p* < 0.001. Mean Index 1 scores were highest in HeW Co-Op (2.32 ± 0.91), followed by Koseiren (2.13 ± 0.93), then Public (1.87 ± 0.93). Post-hoc comparisons showed all pairwise differences were significant (all *p* < 0.001).

Similarly, Index 2 (opportunities to express opinions) scores differed significantly by organizational structure: χ^2^(2) = 267.72, *p* < 0.001. As above, mean Index 2 scores were highest in HeW Co-Op (2.46 ± 0.93), followed by Koseiren (2.21 ± 0.95), then Public (1.96 ± 0.99), and all pairwise differences were significant (*p* < 0.001).

### Associations between worker participation and self-rated health

**Fig. 2:**
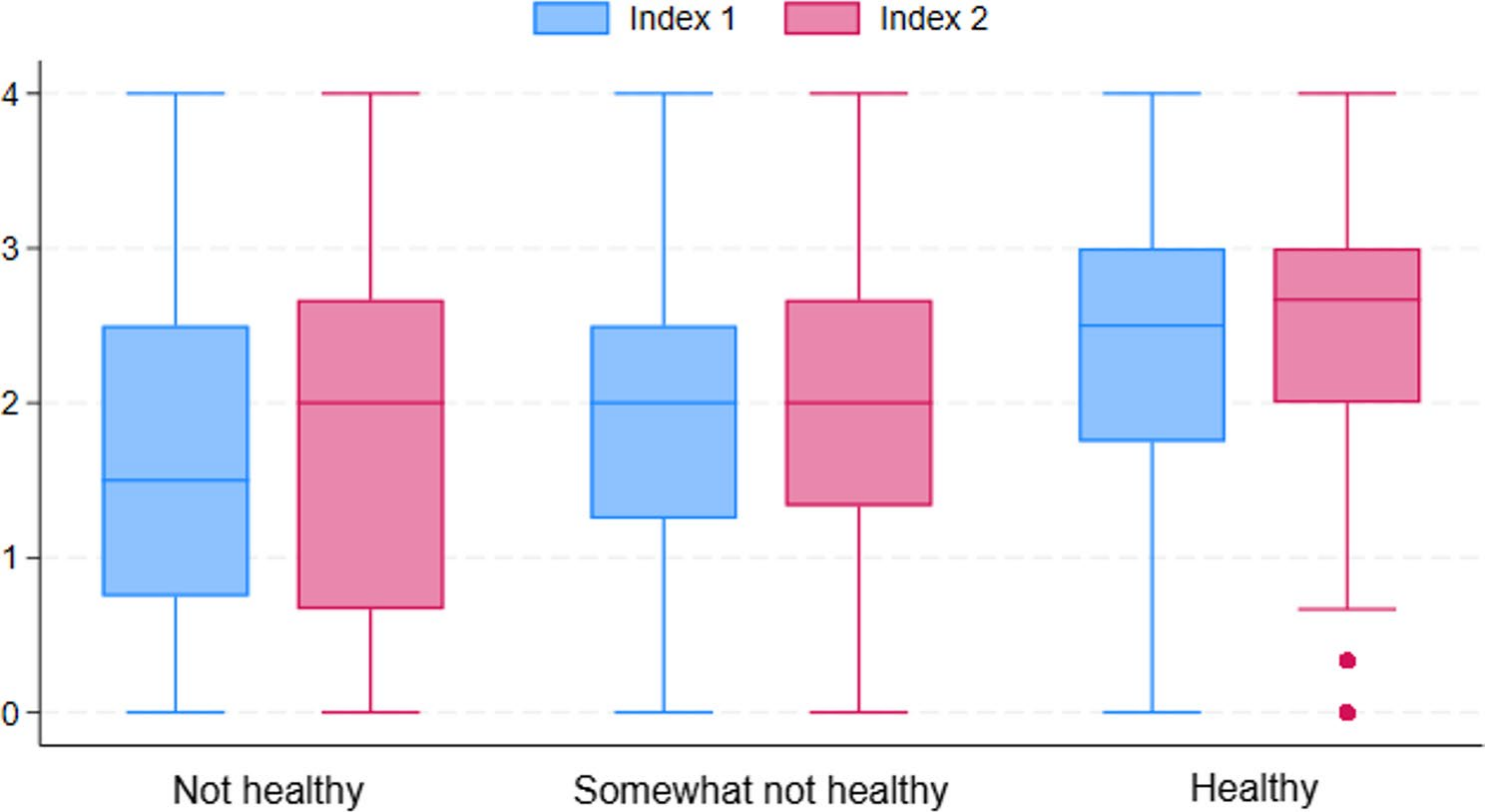
Worker participation scores by self-rated health

Distributions of worker participation scores by self-rated health are depicted in Fig. 2.

The Kruskal-Wallis test indicated that Index 1 (discretion over work) scores varied significantly across worker health ratings: χ^2^(2) = 341.10, *p* < 0.001. Mean Index 1 scores increased with better health ratings: 1.66 (± 1.08) for not healthy, 1.89 (± 0.92) for somewhat not healthy, and 2.31 (± 0.89) for healthy.

Post-hoc tests indicated that mean Index 1 scores were significantly lower for those who identified as not healthy compared to somewhat not healthy (*p* = 0.0126) or healthy (*p* < 0.001), and for those who identified as somewhat not healthy compared to healthy (*p* < 0.001). In unadjusted analysis, a one-point increase in Index 1 scores corresponded with 69% higher odds of better self-rated health (OR = 1.69, 95% CI: 1.60–1.78, *p* < 0.001).

Index 2 (opportunities to express opinions) scores also varied significantly across worker health ratings: χ^2^(2) = 329.50, *p* < 0.001. Again, mean Index 2 scores increased with better health ratings: 1.72 (± 1.17) for not healthy, 2.00 (± 0.96) for somewhat not healthy, and 2.42 (± 0.92) for healthy. Post-hoc comparisons revealed that mean Index 2 scores were significantly lower for those who identified as not healthy compared to somewhat not healthy (*p* = 0.0178) or healthy (*p* < 0.001), and for those who identified as somewhat not healthy compared to healthy (*p* < 0.001). Each one-point increase in Index 2 score was associated with 64% higher odds of better self-rated health (OR = 1.64, 95% CI: 1.56–1.73, *p* < 0.001).

### Self-rated health as a function of worker participation and organizational structure

Multilevel ordinal logistic regression showed that worker participation and organizational structure predicted self-rated health among Japanese hospital employees better than a null model: χ^2^(16, *N* = 6,309) = 604.44, *p* < 0.001. Adjusted odds of better self-rated health increased by 38% per one-point increase in Index 1 (discretion over work) scores (OR = 1.38, 95% CI = 1.28–1.49, *p* < 0.001) and by 49% per one-point increase in Index 2 (opportunities to express opinions) scores (OR = 1.49, 95% CI = 1.39–1.60, *p* < 0.001). The variance of Index 1- and Index 2-health slopes by organizational structures was 0.00002 and 0.00009, respectively. Approximately 0.4% of variability in self-rated health was attributable to organizational structure before adjusting for worker participation and covariates (ICC = 0.004, 95% CI = 0.001–0.027).

## Discussion

This study explored how three distinct organizational structures influenced worker participation and, in turn, the perceived health of Japanese hospital employees. There were three key findings. First, worker participation – comprising discretion over work and opportunities to express opinions – varied significantly by organizational structure, with higher participation in more cooperative settings. Second, worker participation was positively and significantly associated with self-rated health. Third, despite variations by organizational structure, worker participation maintained a consistent positive association with employee health across settings. Participation is a multidimensional concept comprising distinct practices with differing implications for worker outcomes [34]. The indices used in this study capture discretion over work and opportunities to express opinions, which correspond most closely to participation in work decisions and consultative participation, respectively, according to Cotton et al.’s foundational typology. These do not represent all forms of participation, nor shared organizational governance; however, these forms of participation are characterized as relatively formal, direct, and long-term and are among the most salient to workers’ daily experiences in this setting. The first finding – that organizational structure shapes worker participation – aligns with prior research [6, 9, 12] and supports Karasek’s hypothesis linking decision latitude (participation) to organizational authority structure [4]. Notably, participation was highest in HeW Co-Op, characterized by a cooperative governance model, and lowest within Public, which operate under more centralized, hierarchical governance structures. This mirrors Essex et al.’s finding that hierarchical healthcare organizations tended to limit employee voice [9]. Respondent characteristics differed across organizational structures, but these differences did not consistently align with the observed participation gradient. For example, respondents in the HeW Co-Op tended to be older—a characteristic often associated with higher participation—yet this group also included higher proportions of care workers and part-time employees, who generally reported lower participation. If compositional differences were the primary driver of participation patterns, we would expect respondent characteristics to track more closely with the observed gradient across organizations. Moreover, the final models adjusted for key demographic and work-related factors, reducing the likelihood that observed differences are solely attributable to respondent composition. These results suggest that the governance philosophy, community engagement, and service orientation intrinsic to each structure influence employees’ real or perceived power and control within the organization. Supporting this interpretation, qualitative data from McGlinchey et al. [14] revealed that organizational hierarchy, governance, and feeling valued and supported all impacted employee performance and wellbeing. However, job demands, like workload and mandatory overtime, and rewards, like compensation, also played crucial roles. In line with the JDCS model [11], the observed associations may thus partly reflect differing levels of demand or social support across organizations, factors not measured in this study.

Second, we found that worker participation is positively associated with perceived health among Japanese hospital employees. Specifically, better perceived health was predicted by higher discretion over work and more opportunities to express opinions. This finding is consistent with existing literature [6, 12, 13, 15, 16], but original and important within the Japanese healthcare context, where this relationship has not been explored. Causality cannot be established from this cross-sectional data; therefore, the possibility that better health enables higher participation cannot be excluded. Nevertheless, drawing on Karasek [4] and the JDCS model [11], it is plausible that participation affects work-related and overall wellbeing in such a way that enhances perceived health.

The third key finding was the robust, largely consistent association between worker participation and self-rated health across organizational structures, underscored by the minimal ICC. Organizational structure accounted for very little variance in self-rated health beyond worker participation and demographic and work-related factors, suggesting that the participatory dynamics fostered by organizational culture, policies, and practices - rather than formal structure alone - are central to employee health. The observed gradient in participation across governance models can be interpreted through broader organizational and institutional perspectives. Research on democratic governance indicates that participation embedded within organizational structures, such as worker cooperatives, legitimizes employee agency, diffuses power, and enables broader involvement in decision-making [35], thereby fostering norms that support worker voice, collective problem-solving, and shared trust. Although health and social care cooperatives are heterogeneous, they consistently demonstrate strengthened democratic processes and improved working conditions, which may reinforce participation in everyday practice. In contrast, hierarchical medical organizations may dilute employee voice as concerns move through multiple managerial layers, contributing to frustration with slow or ineffective change and limiting collective responsibility. The participation gradient observed in this study thus likely reflects differences in how governance models translate formal structures into lived opportunities for involvement.

While comparative evidence remains limited, European studies similarly suggest that internal organizational culture - including managerial practices that support employee autonomy and participation - outweighs formal structure in shaping performance and wellbeing [6]. In one of the few Japanese studies addressing these issues, Yamaguchi et al. [19] reported variation in job control (participation) among nurses across diverse care settings linked to turnover intention. While their discussion emphasized job demands, our findings suggest that organizational context and participatory practices also play a meaningful role. Together, these findings highlight an interconnected influence of organizational structure, job demands, and participation on workplace wellbeing and employee health, consistent with the JDC and JDCS models [4, 11].

### Implications

Higher worker participation was associated with better self‑rated health among Japanese hospital employees across all organizational structures. Embedding participatory workplace practices and policies to expand discretion and voice thus offers a practical and scalable strategy to enhance healthcare workforce wellbeing in varied settings. To achieve this, organizations should cultivate climates of trust and openness, ensure strong managerial support, and implement structures that facilitate meaningful employee involvement [3, 16]. Organizations with lower participation scores warrant priority for these initiatives; however, since organizational structure itself accounted for minimal variance in employee health, our findings should not be interpreted as criticism of any specific organization. Rather, we believe worker participation is a modifiable factor within all healthcare organizations. Future research should explore how participatory practices and policies can be optimally tailored, adapted, and implemented in varied settings.

Beyond a pragmatic approach to promoting workforce health, enhancing worker participation promises substantial returns for healthcare organizations. Evidence shows that organizations that foster meaningful participation report higher employee wellbeing and stronger performance outcomes [6, 12, 16]. Moreover, companies that invest in employee health and wellbeing consistently outperform the broader market [36]. Accordingly, strategies that embed participation into institutional culture represent high‑yield, system‑wide interventions beneficial to both employees and employers [2].

Importantly, participation varied systematically by gender, professional role, and tenure. In particular, doctors consistently reported higher levels of discretion and voice, while nurses, care workers, and other support staff reported lower participation across both indices. These patterns have been documented elsewhere [8, 22] and reflect known role-based stratification within healthcare organizations, where professional status and proximity to decision-making shape access to autonomy and voice, and may be further influenced by intersections among these characteristics, including the predominance of women in nursing [37]. Organizational structure may either reinforce or attenuate such hierarchies in everyday practice. This calls for targeted strategies to increase participation among subgroups including women, nurses, and newer employees. Future implementation studies should test interventions designed to boost participation for these groups, and more broadly, with the goal of scaling initiatives that empower all workers as part of comprehensive occupational health programs.

### Strengths and limitations

This study benefitted from high response rates, minimal missing data, and worker participation indices with good internal consistency, supporting the reliability of the findings. However, the use of a single-item measure for self-rated health limited our ability to capture its multidimensional nature (e.g., physical, mental, and social domains), and the discrete response options may have reduced sensitivity to subtle variation. We were also unable to conduct a formal non-response analysis; staff with fewer opportunities to participate in their work may have been less likely to respond, potentially resulting in a modest underestimation of certain challenges. Nevertheless, the large sample size likely captured a broad range of experiences, including those with more limited participation opportunities. In addition, the data were collected more than a decade ago, and we cannot be certain whether or how organizational contexts and participation practices have changed since that time, thought the aging population in Japan most likely has exacerbated the pressure and costs in the Japanese healthcare sector [38, 39]. Nonetheless, the theoretical mechanisms linking organizational structure, participation, and worker health are likely to remain relevant, and future studies using more recent data could help elucidate temporal shifts in these relationships. Finally, the cross-sectional design precludes causal inference, and future research should clarify the directionality of the association between participation and health and explore underlying mechanisms.

## Conclusion

This study demonstrates that worker participation among Japanese hospital employees varies across organizational structures, with higher participation in cooperative governance models and lower participation in the more hierarchical public hospitals. Crucially, greater worker participation was consistently linked to better self-rated health regardless of organizational structure. These findings underscore the potential for healthcare organizations to promote workforce health by investing in participatory work environments. Interventions aimed at enhancing employee discretion and voice should be prioritized, especially in organizations and among subgroups with lower participation, such as nurses, women, and newer employees. Embedding such participatory practices holds promise not only for improving healthcare workforce wellbeing but also for strengthening organizational performance and sustainability.

## Supplementary Information

Below is the link to the electronic supplementary material.

Supplementary Material 1

## Data Availability

The datasets used and analyzed during the current study are available from the corresponding author on reasonable request.

## Abbreviations

HeW Co-Op: Japanese health and welfare co-operative federation
ICC: Intraclass correlation coefficient
JDC: Job demand-control (model)
JDCS: Job demand-control-support (model)
