# Supplementary material for: Organizational structure, worker participation, and health: a cross-sectional survey study among Japanese hospital employees
Source: BMC Health Serv Res. 2026 May 25;26:1003. doi: 10.1186/s12913-026-14786-7 (PMC13386781; doi:10.1186/s12913-026-14786-7)
Supplement: Supplementary file 1 — Supplementary Material 1 [file 12913_2026_14786_MOESM1_ESM.docx]

**Supplementary File 1: Worker Participation Indices**

Index 1: Discretion over work (Cronbach’s α = 0.855)

How much discretion do you have over your work? Please circle **one** for each.

| Disagree | Disagree somewhat | Neither agree or disagree | Agree somewhat | Agree |
| --- | --- | --- | --- | --- |

1. You are able to adjust your work load
2. You can work at your own pace
3. You have control over the order and manner in which you perform certain tasks
4. You are given sufficient authority to fulfil your duties

Index 2: Opportunities to express opinions (Cronbach’s α = 0.912)

Do you agree with the following statements about your workplace? Please circle **one** for each.

| Disagree | Disagree somewhat | Neither agree or disagree | Agree somewhat | Agree |
| --- | --- | --- | --- | --- |

1. Sufficient structures and opportunities are in place for expressing your opinions

2. Your suggestions and opinions are reflected

3. It's easy to express your opinions

| **Supplementary Table 1** *Worker Participation Scores by Sample Characteristics* | | | | | | | |
| --- | --- | --- | --- | --- | --- | --- | --- |
|  | Index 1 | |  |  | Index 2 | |  |
| Characteristic | *M* | *SD* | *p* |  | *M* | *SD* | *p* |
| Sex |  |  | <0.001 |  |  |  | <0.001 |
| Female | 2.11 | 0.93 |  |  | 2.22 | 0.97 |  |
| Male | 2.33 | 0.93 |  |  | 2.44 | 0.94 |  |
| Profession |  |  | <0.001 |  |  |  | <0.001 |
| Doctor | 2.59 | 0.90 |  |  | 2.70 | 0.90 |  |
| Nurse | 1.99 | 0.89 |  |  | 2.20 | 0.92 |  |
| Care Worker | 2.17 | 0.93 |  |  | 2.33 | 0.98 |  |
| Administrator | 2.30 | 0.97 |  |  | 2.22 | 1.06 |  |
| Other medical specialist | 2.28 | 0.90 |  |  | 2.34 | 0.94 |  |
| Other support staff | 2.08 | 1.02 |  |  | 2.07 | 0.98 |  |
| Employment status |  |  | <0.001 |  |  |  | <0.001 |
| Full-time permanent | 2.15 | 0.92 |  |  | 2.32 | 0.94 |  |
| Part-time temporary | 2.23 | 0.93 |  |  | 2.25 | 1.00 |  |
| Fixed-term contract | 2.16 | 0.91 |  |  | 2.06 | 1.01 |  |
| Commissioned staff | 1.97 | 1.11 |  |  | 1.92 | 1.02 |  |
| Dispatched staff | 1.73 | 1.01 |  |  | 1.68 | 1.00 |  |
| Current hospital tenure |  |  | 0.004 |  |  |  | <0.001 |
| <1 year | 2.05 | 0.91 |  |  | 2.19 | 0.97 |  |
| 1-5 years | 2.15 | 0.93 |  |  | 2.21 | 0.98 |  |
| 5-10 years | 2.16 | 0.94 |  |  | 2.27 | 0.98 |  |
| >10 years | 2.19 | 0.94 |  |  | 2.33 | 0.95 |  |
| *Note.* Index 1: Discretion over work. Index 2: Opportunities to express opinions. Correlations between age and Index 1 (r = 0.042, p < 0.001) and Index 2 (r = 0.013, p = 0.308). | | | | | | | |

| **Supplementary Table 2** *Self-Rated Health by Sample Characteristics* | | | | | | | | | | |
| --- | --- | --- | --- | --- | --- | --- | --- | --- | --- | --- |
|  | Not healthy | |  | Somewhat not healthy | |  | Healthy | |  |  |
| Characteristic | *n* | % |  | *n* | % |  | *n* | % |  | *p* |
| Sex |  |  |  |  |  |  |  |  |  | 0.676 |
| Female | 165 | 3.1 |  | 1,700 | 32.4 |  | 3,382 | 64.5 |  |  |
| Male | 49 | 3.5 |  | 468 | 33.2 |  | 894 | 63.4 |  |  |
| Profession |  |  |  |  |  |  |  |  |  | <0.001 |
| Doctor | 11 | 3.2 |  | 62 | 18.2 |  | 267 | 78.5 |  |  |
| Nurse | 83 | 3.1 |  | 962 | 35.4 |  | 1,676 | 61.6 |  |  |
| Care Worker | 56 | 5.4 |  | 388 | 37.3 |  | 597 | 57.3 |  |  |
| Administrator | 27 | 2.5 |  | 309 | 28.4 |  | 751 | 69.1 |  |  |
| Other medical specialist | 33 | 2.9 |  | 346 | 30.6 |  | 751 | 66.5 |  |  |
| Other support staff | 9 | 2.4 |  | 123 | 32.4 |  | 248 | 65.3 |  |  |
| Employment status |  |  |  |  |  |  |  |  |  | <0.001 |
| Full-time permanent | 163 | 3.6 |  | 1,557 | 34.5 |  | 2,795 | 61.9 |  |  |
| Part-time temporary | 40 | 2.7 |  | 421 | 28.3 |  | 1,028 | 69.0 |  |  |
| Fixed-term contract | 7 | 1.8 |  | 117 | 30.2 |  | 264 | 68.0 |  |  |
| Commissioned staff | 7 | 3.9 |  | 65 | 35.9 |  | 109 | 60.2 |  |  |
| Dispatched staff | 0 | 0.0 |  | 25 | 23.1 |  | 83 | 76.9 |  |  |
| Current hospital tenure |  |  |  |  |  |  |  |  |  | <0.001 |
| <1 year | 20 | 2.6 |  | 185 | 24.5 |  | 551 | 72.9 |  |  |
| 1-5 years | 50 | 2.3 |  | 611 | 28.3 |  | 1,495 | 69.3 |  |  |
| 5-10 years | 45 | 3.0 |  | 484 | 32.7 |  | 949 | 64.2 |  |  |
| >10 years | 101 | 4.4 |  | 905 | 39.6 |  | 1,280 | 56.0 |  |  |
|  |  |  |  |  |  |  |  |  |  |  |
|  | *M* | *SD* |  | *M* | *SD* |  | *M* | *SD* |  |  |
| Age (years) | 42.4 | 11.4 |  | 42.2 | 12.0 |  | 40.3 | 12.1 |  | <0.001 |
| *Note. N* = 6,732. | | | | | | | | | | |
